# Serum MACC-1: a new biomarker for breast cancer

**DOI:** 10.18632/oncotarget.27813

**Published:** 2020-12-01

**Authors:** Meraj Ahmed, Mohammad Aslam

**Affiliations:** ^1^Department of Surgery, JN Medical College, Aligarh Muslim University, Aligarh, UP, India

**Keywords:** serum MACC-1, MACC-1, new biomarker, breast cancer

## Abstract

Metastasis-associated in colon cancer-1 (MACC-1) is a newly identified tumor marker, found to express in various normal and cancerous tissue. This study is conducted to evaluate the serum MACC-1 level as a diagnostic marker for breast cancer (BC). Sixty new BC patients were included in this study. Patients who received neoadjuvant chemotherapy or with metastatic disease were excluded. Eighty patients of benign disease were taken as control group. All the patients were females with the mean age of 46.7 ± 10.6 years in study group and 40.2 ± 8.4 years in control group (*p* = 0.0001). The mean serum MACC-1 level in BC patients was 3.46 ± 1.3 ng/ml which was significantly higher than control mean serum MACC-1 level (1.90 ± 0.2 ng/ml) (*p* < 0.0001). On ROC analysis, the AUC was 0.98 (*p* ≤ 0.0001; 95% CI = 0.97–1.0) i.e., a good predictor for breast cancer. At the cut-off value of 2.12 ng/ml, the sensitivity and the specificity of serum MACC-1 were 96.7% and 92.5%, respectively. This study showed that serum MACC-1 can be a potential biomarker for diagnosis and tumor progression in patients with breast cancer.

## INTRODUCTION

Breast cancer is the most commonly diagnosed cancer and the leading cause of cancer death among females [1]. Although the outcome of the localized breast cancer patients has improved over time, the survival of patients with metastatic disease (stage IV) remain dismal with five-year survival rate of only 27% [2, 3]. However in the developing nations the survival of patients with breast cancer is still low, owing to the advanced stage at presentation [4]. The outcome of the breast cancer patients can be greatly improved by early detection of the disease coupled with effective treatment [5].

There are various tumor markers to screen, diagnose or predict the outcome for breast cancer. Of these only ER, PR and Her 2 markers are routinely used. However markers like CEA, CA 15-3 and CA 27.29, p53, Ki67 etc. are not recommended in clinical practice because of low sensitivity and/or specificity [6]. Various serum protein and mRNA based markers are being studied to diagnose and prognosticate the patients with breast cancer. These marker tests are non-invasive and also have shown promising results but require additional validation to confirm the clinical value of these test [6].

Metastasis-associated in colon cancer-1 (MACC-1) is a newly identified tumor marker, first identified in colon cancer tissue as a prognostic indicator and inducer of metastasis [7]. The expression is high in malignant tissues than normal tissues and tumors with metachronous metastasis express significantly higher MACC-1 mRNA levels than tumors that have not metastasized [8]. The MACC-1 gene is located on human chromosome 7p21 and regulates hepatocyte growth factor HGF-MET pathway – a key part in cellular growth, angiogenesis, invasiveness and metastasis [8]. It is also found to express in other normal and cancerous tissue of gastrointestinal tract, pancreas, ovary, breast, pituitary gland, kidney, lung, bone marrow etc [7]. The expression of MACC-1 in breast cancer and its correlation with outcomes is little known. This study is therefore conducted to evaluate the serum MACC-1 level as a diagnostic marker for breast cancer.

## RESULTS

### Clinico-pathological profile of the patients

The study group included 60 patients of carcinoma breast in various stages. All the patients were females with the mean age of 46.7 ± 10.6 years. The maximum number of the patients were in the age group of 31–50 years and most of them were in pre-menopausal state (58.3%). Maximum number of patients were in TNM stage III (60%) and had Nottingham grade II tumor (61.7%). The lymph node, and the receptor status are given in Table 1. The control group included 80 female patients of other benign diseases with mean age of 40.2 ± 8.4 years (Table 1).

**Table 1:** Clinico-pathological profile of the patients

Variables	Status	Study group (*n* = 60)	Control group (*n* = 80)	*P* value
Age (Mean ± SD) years		46.7 ± 10.6	40.2 ± 8.4	0.0001
Menopause	Yes	25 (41.7%)	16 (20%)	0.005
	No	35 (58.3%)	64 (80%)	
Tumor stage	T1	03 (05%)		
	T2	24 (40%)		
	T3	14 (23.3%)		
	T4	19 (31.7%)		
TNM stage	I	03 (05%)		
	II	21 (35%)		
	III	36 (60%)		
Tumor grade	I	03 (05%)		
	II	37 (61.7%)		
	III	20 (33.3%)		
Lymph Nodes	Positive	50 (83.3%)		
	Negative	10 (16.7%)		
ER	Positive	19 (31.7%)		
	Negative	41 (68.3%)		
PR	Positive	12 (20%)		
	Negative	48 (80%)		
HER 2	Positive	19 (31.7%)		
	Negative	41 (68.3%)		
Serum MACC-1 (ng/ml) (Mean ± SD)		3.46 ± 1.3	1.90 ± 0.2	< 0.0001

### Association between serum MACC-1 levels and clinico-pathological variables

**Figure 1:**
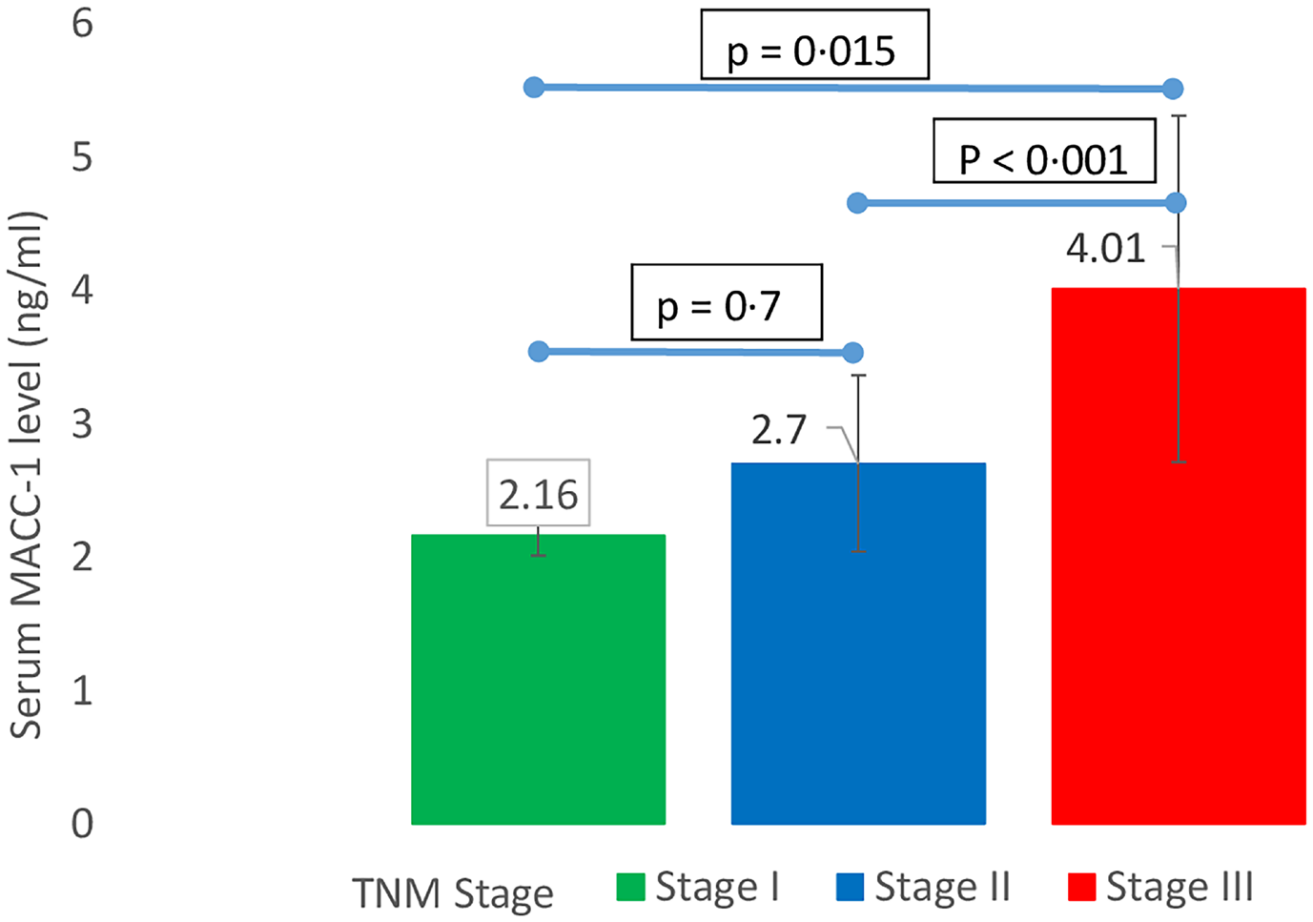
Correlation between TNM stage of breast cancer and serum MACC-1.

**Figure 2:**
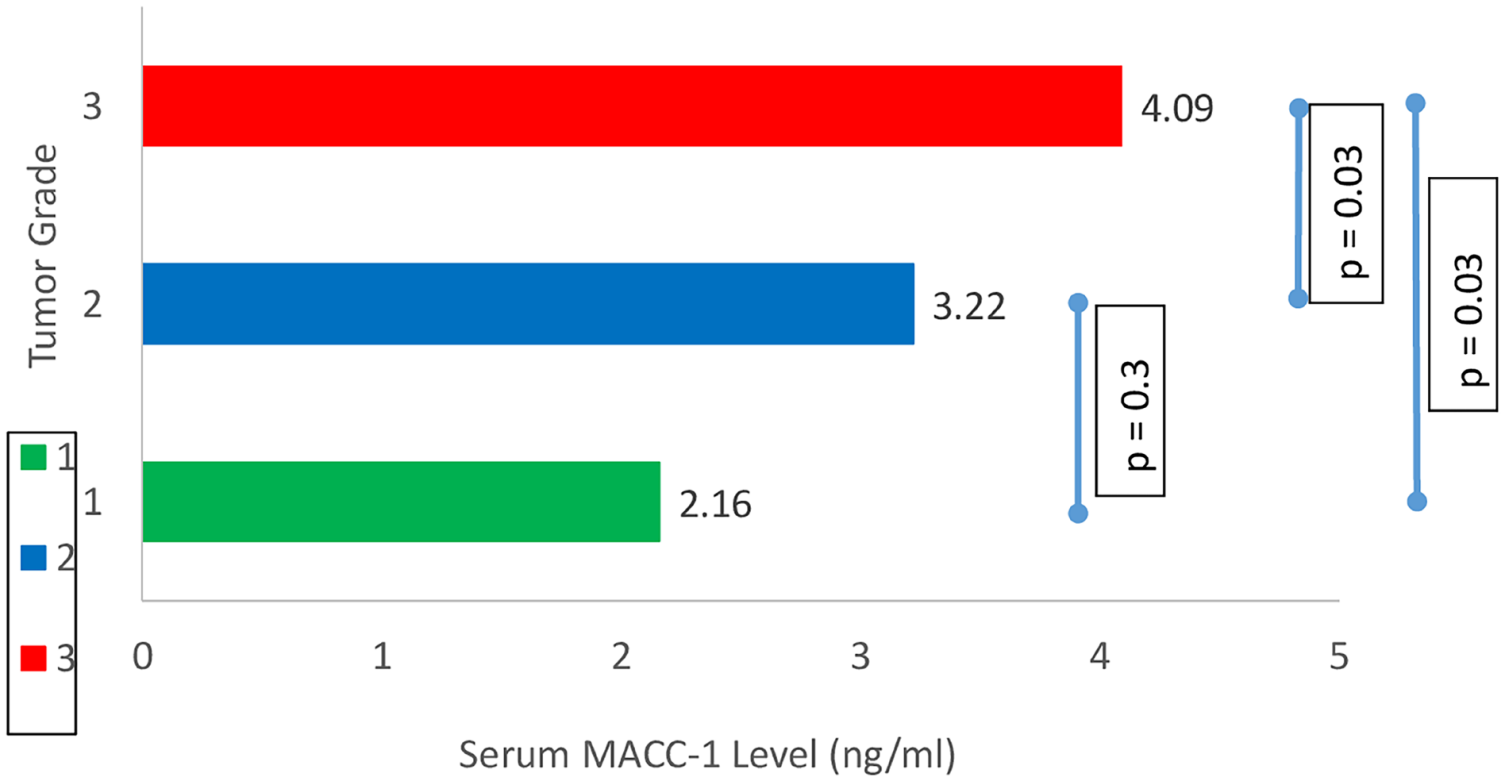
Correlation between tumor grade and serum MACC-1.

The mean serum MACC-1 level in breast cancer patients was 3.46 ± 1.3 ng/ml which was significantly higher than control subjects mean serum MACC-1 level (1.90 ± 0.2 ng/ml) (*p* < 0.0001). In the study group serum MACC-1 level increased with the increasing T-stage of the tumor and the difference was statistically significant. Further serum MACC-1 levels were higher in patients with positive axillary lymph nodes compared to those without nodal disease (3.65 ± 1.29 ng/ml, 2.51 ± 0.33, respectively; *p* = 0.008) (Table 2). Higher serum MACC-1 levels were also observed with increasing TNM stages (*p* < 0.0001) (Figure 1) and tumor grade (*p* = 0.007) (Figure 2). However serum MACC-1 did not correlate with the ER, PR and Her 2 receptor status (Table 3).

**Table 2:** Correlation between serum MACC-1 and T stage and Nodal status of the breast cancer

S. No.	T Stage	Serum MACC-1 (ng/ml) Mean ± SD	*P* value
1	T1	2.16 ± 0.14	
2	T2	2.90 ± 0.69	< 0.001
3	T3	3.43 ± 1.05	
4	T4	4.38 ± 1.47	
5	**Lymph Node Status**		
	LN +	3.65 ± 1.29	0.008
	LN –	2.51 ± 0.33	

**Table 3:** Receptor status and serum MACC-1 level

S. No.	Receptor	Status	Serum MACC-1 level (ng/ml) Mean ± SD	*P* value
1	ER	Positive	3.05 ± 1.34	0.24
		Negative	3.64 ± 1.33	
2	PR	Positive	3.06 ± 1.46	0.40
		Negative	3.55 ± 1.32	
3	HER 2	Positive	3.45 ± 1.37	0.93
		Negative	3.37 ± 1.30	

### Diagnostic value of serum MACC-1 for breast cancer

**Figure 3:**
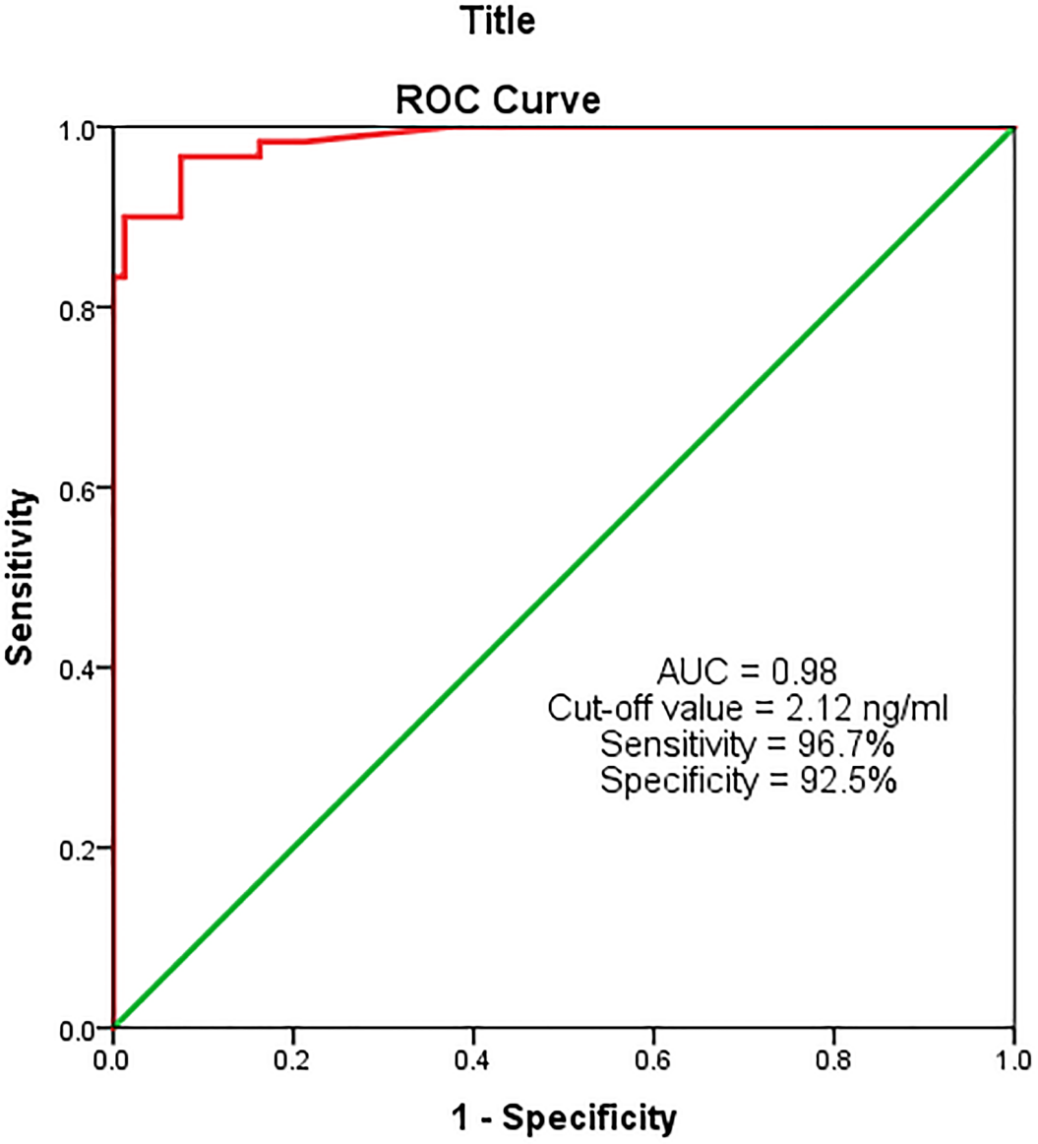
ROC analysis for serum MACC-1 between study and control groups.

On ROC curve analysis of serum MACC-1 in the study and the control groups, the AUC was found to be 0.98 (*p* ≤ 0.0001; 95% CI = 0.97–1.0) i.e., a good predictor for breast cancer. At the cut-off value of 2.12 ng/ml, the sensitivity and the specificity of serum MACC-1 were 96.7% and 92.5% respectively (Figure 3).

## DISCUSSION

The current study is one of the initial study to evaluate the diagnostic and prognostic value of novel serum MACC-1 marker in breast cancer patients. Various other serum biomarkers have been used for the diagnosis of breast cancer but these markers fall short of a good sensitivity and/or specificity [6]. Thus a search for a better serum biomarker remains imperative.

In this study, serum MACC-1 levels were significantly elevated (*p* < 0.0001) in breast cancer patients compared to the control group. Also the ROC analysis showed an AUC of 0.98, signifying a good discriminatory ability of serum MACC-1 marker for breast cancer. At the cut-off value of 2.12 ng/ml this marker has a high sensitivity and specificity (96.7% & 92.5%, respectively). Thus, serum MACC-1 can serve as a diagnostic marker for BC. A similar study by Tan W et al. (2016), showed serum MACC-1 as a diagnostic marker, although with a lower sensitivity and specificity (71.4% & 89.1%, respectively) compared to our study [9].

Our study showed a significant rise in the serum MACC-1 levels with the increasing tumor size, grade and TNM stage. Also a significantly higher serum MACC-1 levels were noted in our BC patients with positive lymph nodes. Thus, a rising serum MACC-1 level may indicate tumor progression. However this marker did not show any significant association with the ER, PR or Her-2 receptor status. These similar findings were also noted by Tan W et al in their study [9]. Another study showed an increased MACC-1 in tumor tissue and its positive correlation with TNM stage, tumor size and nodal status [10].

## MATERIALS AND METHODS

Sixty newly diagnosed breast cancer (BC) patients of stage 0 – III, presented between Jan 2017 and Dec 2018 treated at JN Medical College, Aligarh Muslim University, Aligarh, UP, India were included in this study. Patients who received neoadjuvant chemotherapy or were having metastatic disease at diagnosis were excluded from the study. The diagnosis of breast cancer was based on clinical assessment, radiological investigation and biopsy from the tumor. Patients were staged according to the TNM staging system and were treated according to the standard protocol as per the National Comprehensive Cancer Network (NCCN) guidelines. The patients were followed every 3-6 months with clinical examination, routine investigations and investigations for metastasis if needed. Eighty patients with other benign disease treated at our hospital were taken as control group. The patients in the control group were age matched with study group and had no malignant disease at the time of study or in past.

The blood samples for MACC-1 were taken from both the study and the control groups before starting any therapy. After separation, the serum samples were stored at – 80°C. The tests were performed by double antibody sandwich ELISA technique using Human MACC-1 ELISA Kit (Wuhan Fine Biotech Co., Ltd, Wuhan, Hubei, China) according to the manufacturer’s protocol.

The study was approved by the institute ethics and research advisory committee, faculty of Medicine, Aligarh Muslim University, Aligarh, UP, India. Written informed consent were obtained from participants and the anonymity of their identities were maintained during analysis.

The statistical analysis was performed using the SPSS (version 13.0). Receiver operating characteristic (ROC) analysis was done and area under curve (AUC) was calculated to assess the sensitivity, specificity and the diagnostic power of the serum MACC-1. A *p*-value of < 0.05 was taken as statistically significant.

## CONCLUSIONS

This study showed that serum MACC-1 can be a potential biomarker for diagnosis and tumor progression in patients with breast cancer. Being least invasive and easily detectable, serum MACC-1 can supersede other biomarkers that require tissue sample. Further prospective studies are warranted to validate our findings.

## Abbreviations

MAAC-1: Metastasis-associated in colon cancer-1
BC: Breast cancer
ER: Estrogen Receptor
PR: Progesterone Receptor
HER-2: Human Epidermal growth factor Receptor 2
CEA: Carcino-embryonic Antigen
TNM: Tumor Node Metastasis
NCCN: National Comprehensive Cancer Network
ROC: Receiver Operating Characteristics
AUC: Area under curve
